# Quality improvement priorities for safer out-of-hours palliative care: Lessons from a mixed-methods analysis of a national incident-reporting database

**DOI:** 10.1177/0269216318817692

**Published:** 2018-12-12

**Authors:** Huw Williams, Sir Liam Donaldson, Simon Noble, Peter Hibbert, Rhiannon Watson, Joyce Kenkre, Adrian Edwards, Andrew Carson-Stevens

**Affiliations:** 1 Division of Population Medicine, School of Medicine, Cardiff University, Cardiff, UK; 2 London School of Hygiene & Tropical Medicine, London, UK; 3 Marie Curie Research Centre, Division of Population Medicine, School of Medicine, Cardiff University, Cardiff, UK; 4 Centre for Healthcare Resilience and Implementation Science, Australian Institute of Health Innovation, Macquarie University, North Ryde, NSW, Australia; 5 University of South Wales, Pontypridd, UK; 6 Department of Family Practice, University of British Columbia, Vancouver, BC, Canada

**Keywords:** Palliative care, patient safety, primary care, quality improvement

## Abstract

**Background:**

Patients receiving palliative care are often at increased risk of unsafe care with the out-of-hours setting presenting particular challenges. The identification of improved ways of delivering palliative care outside working hours is a priority area for policymakers.

**Aim:**

To explore the nature and causes of unsafe care delivered to patients receiving palliative care from primary-care services outside normal working hours.

**Design:**

A mixed-methods cross-sectional analysis of patient safety incident reports from the National Reporting and Learning System. We characterised reports, identified by keyword searches, using codes to describe what happened, underlying causes, harm outcome, and severity. Exploratory descriptive and thematic analyses identified factors underpinning unsafe care.

**Setting/participants:**

A total of 1072 patient safety incident reports involving patients receiving sub-optimal palliative care via the out-of-hours primary-care services.

**Results:**

Incidents included issues with: medications (n = 613); access to timely care (n = 123); information transfer (n = 102), and/or non-medication-related treatment such as pressure ulcer relief or catheter care (n = 102). Almost two-thirds of reports (n = 695) described harm with outcomes such as increased pain, emotional, and psychological distress featuring highly. Commonly identified contributory factors to these incidents were a failure to follow protocol (n = 282), lack of skills/confidence of staff (n = 156), and patients requiring medication delivered via a syringe driver (n = 80).

**Conclusion:**

Healthcare systems with primary-care-led models of delivery must examine their practices to determine the prevalence of such safety issues (communication between providers; knowledge of commonly used, and access to, medications and equipment) and utilise improvement methods to achieve improvements in care.

**What is already known about the topic?**

- Around 2%–3 % of consultations in primary care are prone to patient safety incidents.
- Patients receiving palliative care are not immune to patient safety concerns.
- ‘Out-of-hours’ services are responsible for providing care for two-thirds of the working week (18:30 to 08:00 on weekdays, and all hours at weekends in the United Kingdom).

**What this paper adds?**

- Target patient safety issues for improving palliative care in the out-of-hours setting include medication provision, timely access to care and non-medication treatments such as catheter care and information transfer between providers.
- Harm outcomes commonly include pain, emotional distress, unnecessary hospital admission, and hastened death.

**Implications for practice, theory, or policy**

- Interventions to address frequently identified sources of harm are presented and should be evaluated robustly in future implementation studies.

## Introduction

Since the publication of the Institute of Medicine’s report ‘*To Err is Human*’ in 1999, healthcare services worldwide have endeavoured to reduce the burden of unsafe healthcare.^1^ In 2004, the World Health Organization (WHO)^2^ launched the World Alliance for Patient Safety to advance the patient safety agenda with the goal of reducing the adverse effects of unsafe healthcare. Palliative care is not exempt from these risks. More recently, the Universal Health Coverage draws attention to how all people and communities should have access to high-quality, safe palliative care.^3^

Palliative care, increasingly delivered in community settings, poses unique patient safety challenges.^4,5^ In many countries, despite out-of-hours (OOH) services providing care for almost two-thirds of the week (18:30 to 08:00 on weekdays, and all hours at weekends in the United Kingdom), most resources go to in-hours services. As many as 30% of patients have contact with the OOH service in the last days of life.^6^ In the United Kingdom, a range of healthcare professionals, with variable training in end-of-life care practices, are required to meet a patient’s changing needs around the clock. They often lack consistent access to clinical information.

An estimated 2–3 of every 100 consultations in primary care result in a patient safety incident, 4% involving serious harm.^7^ Past studies of palliative-care safety have focussed almost entirely on hospice- or hospital-based care. These have identified concerns about the management of pressure ulcers and the safe use of syringe drivers.^8–11^ Despite patients’ known preferences to receive end-of-life care in their own homes, this is often not achieved.^12,13^ Addressing this challenge will mean primary-care services take a leading role and likely through OOH services.

Investigation of high-profile deaths in the health care system of the United Kingdom have highlighted the complexity associated with providing palliative care in the OOH context.^14^ Care is delivered by many different providers, unfamiliar with a patient’s medical history or current needs and wishes, with many consultations occurring over the telephone, often without face-to-face contact.^15^ A clear understanding of the sources of unsafe care has emerged as a top research priority for patient groups and policymakers.^16^

The analysis of patient safety incident reports can provide valuable insights into healthcare-associated harm.^17^ Such analysis has advanced research into the scope for safer primary care^18,19^ as well as in identifying systemic causes of harms in hospital settings.^20^ With primary-care safety emerging as a global priority for policymakers and increasing focus on delivering palliative care in patients’ homes,^21–23^ a better understanding of risks and causes of harm in this complex area is badly needed.

To provide a foundation for healthcare systems and organisations to prioritise their improvement, we analysed palliative care–related patient safety incidents, reported to a national database, to derive insights into the nature and causes of unintended harm.

## Methods

We carried out a cross-sectional, mixed-methods study of a patient safety incident database. This combined a detailed data coding process and iterative generation of data summaries using exploratory descriptive (quantitative) analysis and thematic (qualitative) analysis methods.^19^

### Data source

Data were extracted from the National Reporting and Learning System (NRLS).^24^ This is a database of over 15 million patient safety incident reports from healthcare organisations in England and Wales. A patient safety incident is defined as, ‘any unintended or unexpected incident that could have harmed or did harm a patient during health care delivery’.^25^ Reporting began voluntarily in 2003, but since 2010, it has been mandatory to report any incident that resulted in severe patient harm or death. Healthcare professionals submit reports to their local healthcare organisations, where the reports are first analysed and anonymised and then submitted in batches to the NRLS. Reports contain structured information about location, patient demographics, and the reporter’s perception of harm severity, augmented by unstructured free-text descriptions of the incident, offering granular detail about potential contributory factors and planned actions to prevent reoccurrence. The database was described in more detail in a study of patient safety–related hospital deaths in England.^20^

### Study population

The study period was between 1 January 2009 and 30 September 2013. We searched the free-text fields of patient safety incident reports submitted from primary-care services (n = 240,000) using keywords to identify records related to palliative care (n = 7413; Appendix 1). The search was then refined to extract those relating to OOH care (n = 2502; Appendix 2).

**Figure 1.**
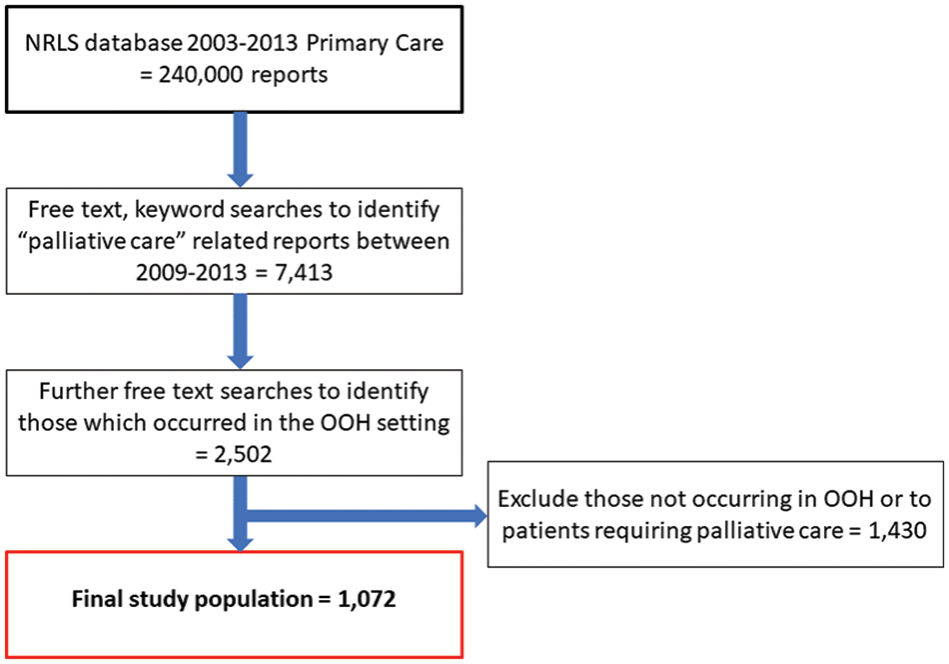
Flowchart of sample identification.

Reports found not to be describing patient safety incidents, incidents not involving palliative care, or not occurring in OOH settings (n = 1,430) were excluded following manual review by two clinical researchers (H.W. and R.W.). The resulting study population comprised 1072 patient safety incident reports (Figure 1).

### Data familiarisation and coding

A classification system, aligned with the WHO International Classification for Patient Safety,^25^ was developed using an inductive grounded approach,^26^ incorporating multiple coding frameworks. These frameworks were empirically developed in-house using an inductive grounded approach, over a period of several months. A primary-care-specific classification system was developed in order to reflect the unique challenges of patient safety in this setting. Codes were developed based on the types of incidents identified in the reports, following discussion within the coding team, which comprised physicians and patient safety experts.^27^ H.W. and R.W. were trained in root cause analysis and human factors in healthcare and reviewed the free-text component of each incident report. They coded the information in relation to the following: the primary patient safety incident type that was reported to have directly affected patient care (e.g. wrong dose of diamorphine administered), the chain of incidents leading up to the incident (e.g. error in setting up rate of syringe driver delivery); the contributory factors (e.g. staff knowledge); and reported patient harm outcomes. A random sample of 20% of reports were double-coded to ensure consistent interpretation of codes and definitions. Difficult cases were discussed at regular team meetings and a third senior investigator (A.C-S.) arbitrated.

### Generation of data summaries and identification of themes

We undertook an exploratory descriptive analysis to assess the most frequent and harmful incident types, the associated chain of incidents, and contributory factors. We used thematic analysis to identify and describe recurring themes that could be targeted to mitigate future similar incidents. The most commonly identified causes were identified as priority areas for improvement and potential interventions, suggested by the reporter, identified by literature searches or the experience of the team, were summarised in a driver diagram. This is a quality improvement tool to highlight priority areas for change, by mapping the relationships between a project aim and key areas in a system for intervention.^28,29^ We conducted focussed literature searches to identify existing initiatives for promoting patient safety in each priority area. Where available, the strength of each intervention was graded using the US Department of Veterans Affairs classification, where the strongest designs are permanent and physical (e.g. forcing functions around medication prescribing) rather than temporary and procedural (e.g. awareness raising communications around prescribing safety).^30^

## Results

Almost two-thirds (n = 695, 64.8%) of the patient safety incidents that comprised the study population of 1072 reports described actual harm to patients and the remaining reports described potentially harmful events (Table 1). Serious harm (moderate harm or worse) occurred in 129 (12%), resulting in hospital admission, permanent injury or death. There were four main categories of harmful incidents: medication-related issues such as errors in prescribing, dispensing, or administering of medications (n = 618, 66% harmful); delays in access to timely care or advice (n = 123, 65% harmful); shortfalls in the safety of non-medication-based treatment such as catheters and nasogastric tubes (n = 102, 69% harmful); and deficient information transfer across the healthcare boundaries (n = 102, 64% harmful). Cohen’s kappa statistic of inter-coder reliability for primary incident type was high (k = 0.7).

**Table 1.** Harm severity n (% of incident type).

Incident type	No Harm occurred	Low Harm	Moderate Harm	Severe Harm	Death	Harm severity unclear	Total
Medication related	71 (11%)	355 (57%)	45 (7%)	3 (0.5%)	5 (0.8%)	139 (22%)	618
Access to timely care	5 (4%)	63 (51%)	14 (11%)	–	3 (2%)	38 (31%)	123
Information transfer	14 (14%)	53 (52%)	12 (12%)	–	–	23 (23%)	102
Treatment (non-medication)	10 (10%)	51 (50%)	18 (18%)	–	1 (1%)	22 (22%)	102
Other	17 (13%)	44 (35%)	21 (17%)	2 (2%)	5 (4%)	38 (30%)	127
Totals	117	566	110	5	14	260 (24%)	1072

### Medication-related incidents

More than half of the reports described incidents related to medication (n = 618, 58% of all reports). One-third (n = 199) described problems with timely access to medication. Problems with the task of giving the medicine to a patient was mentioned in one-fifth of reports (Example 1, Table 2; n = 124) with nearly one-quarter (n = 28) of these describing failure to administer essential medication resulting in patient distress or pain. A total of 93 reports described prescribing, and a further 70 described dispensing errors, with the wrong drug or wrong strength of drug being prescribed or dispensed. About 75 reports described the use of, or provision of, a syringe driver device as being pivotal to the incident.

**Table 2.** Examples of free-text descriptions and harm severities.

Free-text description	Harm severity
Medication related	
Example 1. Staff nurse reflected on the incident, and she stated that she administered a dose of hyoscine which was prescribed for a syringe driver–1.2 mg–as a stat dose. She realised after giving the injection. She contacted out-of-hours GP, but patient’s breathing worsened and she called 999 after giving adrenaline. No ‘Do not Resuscitate’ order was found in the patient’s home, and so CPR was performed by the ambulance crew. At [time] I contacted A&E and spoke to Sister in Resus. She informed me that the patient was critical and called back [30 min later] to state that the patient was ventilated … A + E sister informed me that patient was transferred to a ward. Ward sister stated that they were trying to send patient home as this was their wish. Telephone call received from Dr [Staff Name] at GP OOHs stating that the patient had died.	Death
Example 2. Patient assessed as being in last few hours of life was prescribed morphine sulphate injection. Primary-Care Trust has ‘End of Life Care (EOLC) Medication’ scheme in place, whereby pharmacy is paid to always keep agreed list of medication in stock. Patient’s relative phoned the pharmacy but was told it was not in stock. DN then phoned. He was also told not in stock. Spent several hours trying to obtain medication – eventually did so from another pharmacy. Further enquiry on Monday revealed that pharmacy in question did have the medication in stock but have internal policy of not dispensing controlled drugs at weekend except under the EOLC scheme and did not identify that this is a palliative-care patient.	Low
Access to timely care	
Example 3. Call received by the night service at [time] to visit a palliative patient in pain. The night service was unable to respond, as all teams were with a patient. The night team attempted to contact the [nursing team] 2 h later in 10 min intervals. They were unable to contact anyone [for three-and-a-half hours] when a member of the day team answered the phone and explained the phone had not been diverted.	Low
Example 4. The patient was dying at home. Patient had injectable medication in-house for control of symptoms. Patient began vomiting at [time]. Patient husband called overnight nursing service [around 1 h later] as he had been told they could visit to give injection of anti-emetic. A member of staff told him they would seek advice and call back. They informed the patient’s husband that they were not able to help and to call [the GP OOH provider]. Patient’s husband called and explained patient was vomiting coffee ground vomit. Was advised to give omeprazole and or Gaviscon. [Name of provider] did not visit. Patient continued to vomit overnight. When staff arrived the next morning, the patient had had several large [episodes of haematemesis] overnight and melaena. Her husband had not been able to clean this himself.	Moderate
Example 5. Called to see patient who is in pain and under palliative care. Patient in pain and very distressed heading towards last days of life. Passed over to unplanned care department at [time]. [3 h later] GP still had not visited. Contacted unplanned care at this time and they said they had a busy night and that the GP who [had just stated work] would see the patient first.	Moderate
Information transfer	
Example 6. Patient’s wife phoned out-of-hours service at [time], and GP decided that a syringe driver needed to be put up. OOH failed to contact DN with appropriate information regarding the patient and at the right time. Insufficient time span for adequate provision of care with a patient at end stages of life. Inappropriate use of DN time.	Unclear
Treatment related	
Example 7. I was contacted by the staff nurse on duty from a nursing home stating that a palliative patient’s catheter was by-passing yesterday and the staff nurse on duty had removed the catheter and not replaced it. The patient had now not passed urine for over 10 h and was in discomfort and pain. I asked the staff nurse why the catheter was not reinserted yesterday as this patient was known to have [a type of cancer] and suffer with retention – that was why the catheter was in place. The staff nurse on duty stated she was unsure why it was not reinserted. I stated that the patient did have all the equipment as I had only done a prescription for them the beginning of the week. I advised the staff nurse to reinsert the catheter; however, she informed me that she was not trained to do catheters and could not perform the procedure. I contacted the DN team covering the nursing home and discussed with the DN in charge, and as this patient is a nursing home patient and because of his [type of cancer], they were not happy to re-catheterise this gentleman. I therefore had no other alternative but to admit this patient to surgical assessment unit at [organisation name] for re-catheterisation. This took me approximately 90 min having to liaise with the SHO on call writing a referral letter and organising the ambulance. Meanwhile this patient was in pain.	Moderate
Example 8. Patient has advanced [neurological disease] and can only communicate using eyes. Feeding tube balloon collapsed, so [the patient] couldn’t have any feed or medication. Has Type 1 diabetes and had had rapid acting insulin but no feed. Despite myself and the GP calling ahead to the surgical registrar, F1 and A&E sister to ensure there would be somebody who could change this tube, the patient went into A&E and nobody was able to change it. The A&E doctor taped it up and sent him home. The tube could not be used, as it could have been misplaced, and he was at risk of aspiration infection etc. This had happened before – exactly the same scenario. After the weekend, we tried to get this changed but was told the radiographer was the only person that could change it and he was too busy this week. The patient was offered admission for NG tube feeding but declined. The appointment was made for 1 week’s time with the radiographer but the tube completely fell out the next day so it would then have had to be completely redone not just changed. Since then, the patient has decided to decline all active treatment and feeding and has gone into a hospice indefinitely.	Severe
Example 9. Elderly patient … at home was being treated for sub-acute bowel obstruction. Despite maximal treatment via syringe driver including octreotide, hyoscine butylbromide, haloperidol, and morphine, [the patient] experienced a gradual accumulation of GI fluid every 48 h which resulted in severe pain … Palliative-care team advised use of a Ryles tube on free drainage. In evening of [date] after visiting, I requested that the DNs insert the tube. After a period of confusion (staff were under impression that he had to go to hospital to have a tube inserted) … it became apparent that the nurse on duty did not feel they had the competency to insert any form of NG tube … The patient did not get a drainage tube at any time that evening, he eventually vomited but remained agitated throughout his last night. Why was no nurse with this basic competency on duty? Does the trust have a policy for this basic nursing procedure in line with the document appended?	Low

GP: general practitioner; CPR: cardiopulmonary resuscitation; OOH: out of hours; DN: district nurse; GI: gastrointestinal; NG: nasogastric.

The chain of events leading to medication incidents often included difficulties accessing timely advice/assessments, problems locating supplies of medications (Example 2, Table 2), a faulty syringe driver or inefficient communication processes.

Staff level contributory factors were common and included failure to follow agreed protocols, mistakes due to inattention or a lack of sufficient knowledge or skills. In 25 reports, insufficient staff capacity was highlighted as an underlying reason for an incident.

Patient outcomes included pain, emotional distress for patients and families, and increased work for members of the healthcare team. These incidents led to serious harm (moderate, severe harm, or death) in 48 and to death in 5 cases.

### Access to timely care

Almost 12% (n = 123) of reports described situations where patients or their relatives struggled to get timely access to needed care. An inability to get an appointment with a clinician accounted for half of these (n = 61) and a quarter (n = 33) involving difficulties securing a home visit (Examples 3, 4, and 5; Table 2). Problems accessing telephone advice was a particular barrier (n = 21).

A lack of, or insufficient numbers of staff (n = 24; Example 5, Table 2) was a commonly identified contributory factor. Other staff level contributory factors included failures to follow protocols, inadequate skills, and mistakes or distraction/inattention. Pain, emotional distress, and untimely death were outcomes described.

### Information transfer

A further 10% (n = 102) of reports described issues with ensuring efficient, accurate transfer of information between healthcare teams. This included referrals to other teams not being made, going missing, or being sent to the wrong place (n = 52; Example 6); misunderstandings in verbal communication between teams (n = 27); and information about a patient’s condition not being made available to other teams (n = 20).

Preceding incidents were described in just over half of these reports and mainly involved assessment or triage-related incidents or additional communication/information transfer–related incidents.

Contributory factors included a failure to follow agreed protocols and organisational problems such as insufficient staffing levels or poorly worded protocols. Again, pain, emotional distress, and additional time spent by staff mitigating the harms were described as outcomes.

### Treatment-related incidents (non-medication)

A further 10% (n = 102) of reports described incidents involving treatments such as urinary catheters, pressure ulcer relief, and nasogastric tubes. Reports described insufficient treatment given across the course of a night or weekend (n = 36), significant delays in commencing treatment (n = 36; Example 7) or some treatments not given at all (n = 27).

Preceding incidents identified included information transfer problems, an inability to access a clinician, and equipment-related incidents.

Identified contributory factors include insufficient staff numbers or being overloaded by work, a failure to follow protocols, or a lack of knowledge or training (Example 8 and 9). Outcomes once more included pain, emotional distress, and hospital admission.

### Driver diagram

**Figure 2.**
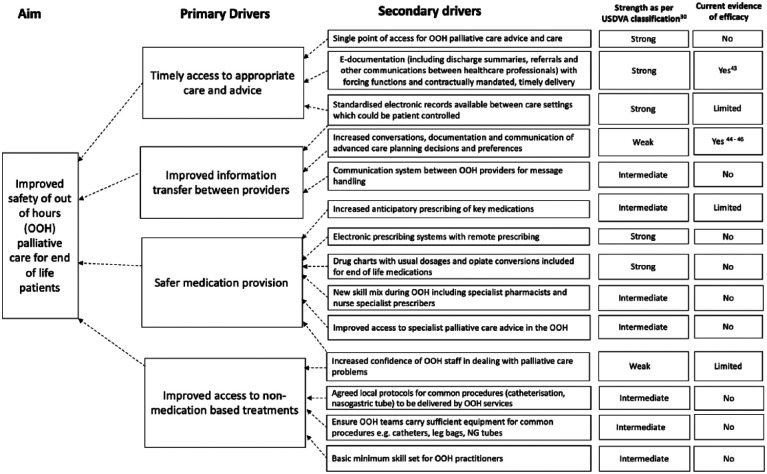
Driver diagram to show potential interventions to improve the safety of out of hours primary care for patients at the end of life.

We mapped our findings to a driver diagram. This showed four main areas of unsafe care, each a primary driver for change.^29^ Focusing on the primary drivers, and drawing on the output of the literature search, the team constructed secondary drivers or interventions which could influence safer care (Figure 2).

## Discussion

### Main findings

We found that unsafe palliative care occurred in four main areas: errors in medication provision; securing access to timely care; inefficient information processes; and non-medication-related treatment provision. Actual harm was a feature of almost two-thirds of patient safety incident reports with many citing emotional and psychological distress to patients, families, and carers.

### Strengths and limitations

Our study is the largest examination of patient safety incidents involving patients requiring palliative care and the first to analyse unsafe care for this group of patients in the OOH primary-care setting. We drew on the largest established national repository of patient safety incident reports in the world to do this.

All incident-reporting systems suffer from under-reporting, to varying degrees. That is why, we have not made inferences about the absolute incidence of different types of harm. It is also important not to generalise too greatly, though it is fair to say that many of our core findings are consistent with studies of harm in other palliative-care settings. Analysis of patient safety incidents allow insights into what happened and why in a way other research methods struggle to do. A focus on the most common incident types and underlying contributory factors, regardless of the level of harm occurring to the patient, allows identification of priority areas for improvement. Our structured process makes sense of large volumes of data. The study team only had access to reports submitted until late 2013; however, by taking 5 years of available data, we have been able to show how an organisation might approach improving palliative care in the OOH primary-care setting. We identified current change options in our more recent focussed literature reviews which complemented our findings, but full systematic reviews may have identified more interventions.

### What this study adds

We found that medication-related incidents were the most commonly reported, in line with other studies of patient safety.^7,17,18,31,32^ The complex dosing regimen and routes of delivery involved led to errors, and the controlled nature of common palliative-care medications led to delays in accessing drugs. Anticipatory prescribing of commonly used drugs, electronic prescribing systems, prescription chart templates, improving skills of staff, and increased access to specialist advice could contribute to reduced delays and safer medication provision (Figure 2).

Improving identification of those requiring anticipatory prescribing could mitigate some of the problems identified and anticipatory prescribing features in most guidelines for end-of-life care.^33^ However, there is a lack of clear evidence that this approach controls symptoms or avoids admission to hospital. Establishing whether it does should be a focus of future research. Our finding of lack of knowledge or skills of clinical staff (both medical and nursing) – whether it be of the medicines themselves or the delivery method – has been demonstrated in other studies.^4,34,35^ Inclusion of palliative care in undergraduate and postgraduate curricula would address this deficit, as would tailored training packages for OOH clinicians who may not have time for extended programmes of study. Over-stretched staff should have access to palliative-care specialist advice quickly, and mechanisms to achieve this need to de designed and evaluated. Pre-populated medicines charts are a way of mitigating prescribing errors; dedicated palliative-care pharmacies – stocked with a locally agreed range of medicines – accessible 24-h a day could further reduce delays in accessing medications.^36^ Specialist pharmacists or nurses with prescribing skills could reduce delays incurred waiting for a doctor to prescribe medications. Communication solutions to enable members of a primary-care team to collaborate effectively – such as encrypted end-to-end messaging systems embedded within the clinical record system – could allow development of a suitable plan for symptom relief without duplicating visits.

A lack of timely access to care from the most appropriate professional led to many delays in care and could be mitigated by patients, families, and carers knowing how to access OOH palliative-care advice, robust triage and prioritisation systems, and electronic messaging systems between healthcare professionals. Development of single point of access for palliative-care patients OOH is being trialled in some areas and should be evaluated robustly for evidence of improved safety outcomes.^37–39^ Triage in OOH services has been highlighted as a cause for concern in several previous studies,^40,41^ particularly how the algorithms used need to be responsive to the needs of patients at the end of life. Once prioritised, OOH services need to ensure a reliable system of communication between the various providers of care and ensure timely attendance.

Information transfer or communication problems often underpinned incidents and are described in other studies of primary-care safety.^17^ Potential interventions could include patient-held unified medical records at the end of life, electronic referrals systems, and robust messaging systems (Figure 2). A unified record of care for patients nearing the end of life, accessible by all those who may need it, should be the goal and could be patient held. This record should include advance-care decisions and patients’ wishes, with record of drug dosages and who to contact if things deteriorate.^42^ These should be electronically based, accessible by all involved agencies and have suitable back up in case of IT-related problems.^43^ Clear protocols of who has responsibility for which aspects of a patient’s care journey should be made available to all providing OOH care.^42^

Advance care planning for those approaching the end of their lives has been promoted as a way of improving care, with advocates suggesting this process could lead to improved identification of palliative patients in triage systems, increased anticipatory prescribing, and reducing unwanted admissions.^44^ Several studies have attempted to increase the frequency and quality of the advance-care planning process, its documentation and communication to OOH services.^45–47^ Successes in this area should be shared widely and adapted to local contexts.

Urinary catheters and pressure ulcers have been major sources of concern for patients and relatives of those nearing the end of life. Staff should have commonly required equipment with them, or within easy reach. Training needs in each organisation should be assessed and addressed – with agreed protocols for certain procedures agreed locally. A dedicated OOH palliative-care team would bridge the skills and knowledge gaps, but its seamless integration with in-hours services is essential. Adding a further team to deliver palliative care must not confuse responsibilities or create communication difficulties.

As new multidisciplinary care models are established, the opportunity to test out such ideas present themselves with the imperative that they are evaluated.^48^

## Conclusion

We have highlighted the nature of actual and potential harms occurring to patients requiring palliative care in OOH settings. Lasting, system-level interventions, particularly those facilitating safe access to medications and treatments, more timely care for those at the end of life and information transfer across care boundaries should be the focus of future improvement initiatives.

## Appendix 1

### Palliative care–related terms

Cyclizine
Ondansetron
Metoclopramide
Levomepromazine
Prochlorperazine
Morphine
Oramorph
Diamorphine
Oxycodone
Oxynorm
Oxycontin
Sevredol
Hyoscine
Midazolam
Diazepam
Lorazepam
Syringe driver
Driver
Pump
Breakthrough
Sub-cutaneous
Sub-cut
Marie Curie
Hospice
Macmillan
EOLCP
Palliative/palliate
End-stage/end stage
EOLCP
Liverpool
Care pathway
End of life
Terminal
Inoperable
DNAR/DNR
Resuscitation
Advance directive
Living will
Advance care planning

## Appendix 2

### Out-of-hours-related terms

Out of hour
On-call
Oncall
On call’
OOH
Duty doctor
Duty dr
Locum
Weekend
Night
Saturday
Sunday
BH
Christmas
Boxing Day
Easter
Holiday
Adastra
Emergency GP
OO hours
Out hours
out - of hours
Out-of-hours
out OH
OofH
Out-hours
Outofhours
Out-of-
O-O-H%
out of – hours
out of-
out off hours
out-off-hours
Oofhours
O of H
out_of_hour
